# Channel Activity of Cardiac Ryanodine Receptors (RyR2) Determines Potency and Efficacy of Flecainide and R-Propafenone against Arrhythmogenic Calcium Waves in Ventricular Cardiomyocytes

**DOI:** 10.1371/journal.pone.0131179

**Published:** 2015-06-29

**Authors:** Eleonora Savio-Galimberti, Björn C. Knollmann

**Affiliations:** 1 Division of Clinical Pharmacology and Oates Institute for Experimental Therapeutics, Department of Medicine, Vanderbilt University School of Medicine, Nashville, United States of America; 2 Division of Cardiovascular Medicine. Department of Medicine, Vanderbilt University School of Medicine, Nashville, United States of America; Loyola University Chicago, UNITED STATES

## Abstract

Flecainide blocks ryanodine receptor type 2 (RyR2) channels in the open state, suppresses arrhythmogenic Ca^2+^ waves and prevents catecholaminergic polymorphic ventricular tachycardia (CPVT) in mice and humans. We hypothesized that differences in RyR2 activity induced by CPVT mutations determines the potency of open-state RyR2 blockers like flecainide (FLEC) and R-propafenone (RPROP) against Ca^2+^ waves in cardiomyocytes. Using confocal microscopy, we studied Ca^2+^ sparks and waves in isolated saponin-permeabilized ventricular myocytes from two CPVT mouse models (Casq2^-/-^, RyR2-R4496C^+/-^), wild-type (c57bl/6, WT) mice, and WT rabbits (New Zealand white rabbits). Consistent with increased RyR2 activity, Ca^2+^ spark and wave frequencies were significantly higher in CPVT compared to WT mouse myocytes. We next obtained concentration-response curves of Ca^2+^ wave inhibition for FLEC, RPROP (another open-state RyR2 blocker), and tetracaine (TET) (a state-independent RyR2 blocker). Both FLEC and RPROP inhibited Ca^2+^ waves with significantly higher potency (lower IC_50_) and efficacy in CPVT compared to WT. In contrast, TET had similar potency in all groups studied. Increasing RyR2 activity of permeabilized WT myocytes by exposure to caffeine (150 µM) increased the potency of FLEC and RPROP but not of TET. RPROP and FLEC were also significantly more potent in rabbit ventricular myocytes that intrinsically exhibit higher Ca^2+^ spark rates than WT mouse ventricular myocytes. *In conclusion*, RyR2 activity determines the potency of open-state blockers FLEC and RPROP for suppressing arrhythmogenic Ca^2+^ waves in cardiomyocytes, a mechanism likely relevant to antiarrhythmic drug efficacy in CPVT.

## Introduction

Type 2 ryanodine receptors (RyR2) are intracellular Ca^2+^ release channels located on the surface of the sarcoplasmic reticulum (SR) in cardiac muscle cells [1–3]. Together with L-type Ca^2+^ channels located on the sarcolemma, RyR2 channels are responsible for the triggering arm of the Ca^2+^-induced Ca^2+^ release mechanism in the ventricle and hence excitation contraction coupling in cardiac cells [4–7]. Mutations in genes encoding the RyR2 channel itself [8], or in genes encoding the RyR2 binding proteins cardiac calsequestrin (Casq2) [9], triadin [10, 11] and calmodulin [12] that regulate RyR2 channel open probability have all been associated with a rare inherited arrhythmia syndrome known as catecholaminergic polymorphic ventricular tachycardia (CPVT). CPVT is characterized by the occurrence of potentially life-threatening polymorphic ventricular tachyarrhythmias in conditions of physical or emotional stress in the absence of structural myocardial changes [8, 9]. Depending on the CPVT mutation, a number of molecular mechanisms have been suggested by different groups: defective SR luminal Ca^2+^ sensing [13], defective inter-domain interaction [14], increased cytosolic Ca^2+^ sensitivity [15], reduced calmodulin binding [16], activation of RyR2 channels by mutant calmodulin [17], among other mechanisms. Regardless of the specific molecular mechanism, CPVT mutations induce a common dysregulation of intracellular Ca^2+^ release that is characterized by an increased open probability of RyR2 Ca^2+^ release channels and results in the generation of arrhythmogenic Ca^2+^ waves [18–20].

We previously reported that the Class IC antiarrythmic drug flecainide (FLEC) inhibits RyR2 channels in the open state, suppresses Ca^2+^ waves and prevents CPVT in mice and humans [21, 22]. The state-independent RyR2 blocker tetracaine (TET) was less effective in suppressing spontaneous Ca^2+^ release in CPVT myocytes [21, 22], suggesting that the state-dependent mode of drug action may contribute to the drug efficacy in CPVT. Here, we hypothesized that differences in RyR2 activity (i.e., due to a CPVT mutation) determine the potency of open-state RyR2 blockers such as FLEC against Ca^2+^ waves in cardiomyocytes. To test this hypothesis, we studied Ca^2+^ sparks and waves in isolated saponin-permeabilized ventricular myocytes from two CPVT mouse models (Casq2^-/-^ and RyR2-R4496C^+/-^), wild-type (WT) mice, and WT rabbits. We find that RyR2 activity determines the potency of open-state blockers FLEC and R-propafenone [RPROP] for suppressing arrhythmogenic Ca^2+^ waves in cardiomyocytes, a mechanism likely relevant to antiarrhythmic drug efficacy in CPVT. Our results also help explain why the contribution of RyR2 block to Ca^2+^ wave suppression by FLEC is more difficult to identify in myocytes with lower Ca^2+^ spark frequency and hence RyR2 open probability than Casq2^-/-^ myocytes [23, 24].

## Materials and Methods

### Experimental procedures and animal models of CPVT

The use of animals in this study was approved by the Animal Care and Use Committee of Vanderbilt University, USA, and performed in accordance with NIH guidelines. Vanderbilt University Review Board specifically approved this study (Animal protocol # M/08/001). Euthanasia of mice and rabbits was performed by heart harvest and exsanguination under surgical plane general anesthesia.

Here we used male Casq2^-/-^ mice that display normal SR Ca^2+^ release and contractile function under basal conditions, but consistently develop ventricular tachycardia during exercise or after catecholamine challenge [25]. We also used male RyR2-R4496C^+/-^ mice generously provided by Dr. S.R. Chen. The R4496C mutation in the mouse model is the equivalent of the R4497C human mutation [26]. The mice are heterozygous for a point mutation in the exon 94 of the RyR2 gene, where cytosine (C) is replaced by thymine (T). As a consequence, the arginine (R) in position 4496 is replaced by a cysteine (C). It has been previously reported that RyR2-R4496C^+/-^ myocytes exhibited enhanced Ca^2+^ sensitivity and, as a consequence of it, increased spontaneous Ca^2+^ waves during electric pacing and isoproterenol challenge [26]. Male c57bl/6 mice were used as a control group (wild-type (WT) group).

Tetracaine, flecainide acetate, and propafenone hydrochloride, as well as all the chemicals used to prepare the solutions (unless otherwise specified) were obtained from Sigma (St. Louis, MO). The stock solutions for all drugs tested were prepared using DMSO as solvent (final concentration 2.5 μl DMSO/ml internal solution). We only tested the effects of R-propafenone (the procedure to separate the enantiomers of propafenone has been described before [18]). The internal solution used as a control solution contained 2.5 μl DMSO/ml (VEH solution). In a separate set of experiments we pre-incubated WT myocytes with caffeine (CAFF) 150 μM to pharmacologically increase the open probability of RyR2 in these cells and test the effect of both open and close channel blockers on Ca^2+^ wave parameters.

Rabbit ventricular myocytes were isolated from hearts of male New Zealand white rabbits (6–7 lbs from Charles River Canada).

In the case of the experiments where we registered Ca^2+^ sparks and waves in mice, we measured ~30–60 cells, N = 2–3 mice/condition studied. In the case of the experiments conducted with rabbit ventricular cardiomyocytes, we measured Ca^2+^ sparks and waves in ~30–40 cells, N = 3 rabbits/condition tested.

### Isolation of ventricular myocytes

Single ventricular myocytes from mice and rabbits were isolated by enzymatic digestion applying a modified collagenase/protease method as previously described. After isolation, cells were washed twice by gravity sedimentation for 20 min in 0.2 mM CaCl_2_ tyrode solution, supernatant removed and cells re-suspended in 0.6 mM Ca_2_Cl DMEM solution (Dulbecco’s Modification of Eagle’s Medium). [Ca^2+^] was progressively increased to the final Ca^2+^ of [1 mM]. The rabbit myocytes used in these experiments were harvested from the free wall of left ventricle.

### Confocal imaging in permeabilized ventricular myocytes

An aliquot of about 200 μl of the solution containing the isolated myocytes was placed in a laminin-coated chamber and allowed to settle down for 6 min before starting the permeabilization. In these chambers, fluid was exchanged in bulk for drug application. The detailed procedure for the chemical permeabilization of mouse cardiac myocytes has been previously reported [18]. The same procedure was used to permeabilize rabbit ventricular myocytes.

The permeabilized cells were imaged using an LSM 510 Zeiss inverted microscope and a 40x oil immersion objective lens (Nikon, Tokyo, Japan). Intracellular fluo-4 was excited at 488 nm with a krypton/argon laser. The fluorescent emission was collected through a long-pass filter (>515 nm). All images were acquired digitally in line-scan mode with 0.2 μm and 0.2 ms per pixel resolution. We only collected data from isolated myocytes that showed the classic brick-shape morphology. For each drug concentration, 7 to 10 microscope fields were examined. Wave incidence was calculated as the fraction of cells waving divided by the total number of brick-shaped myocytes per field.

### Ca^2+^ spark and Ca^2+^ wave data analysis

In all cases, fluorescence images were analyzed using ImageJ, the public domain NIH Image program (developed at NIH and available on the internet at http://rsb.info.nih.gov/nih-image). Ca^2+^ sparks and waves were acquired using the confocal microscope in the line-scan mode. The scan line was placed parallel to the longitudinal (main) axis of the myocyte. LS were obtained only from the population of waving cells. For the purpose of the wave analysis in the present work, only propagated Ca^2+^ waves were analyzed. Wave amplitude, frequency, and propagation speed were also measured for each drug concentration and concentration-response curves (CRCs) constructed for each wave parameter. The corresponding IC_50_ was obtained by fitting the CRCs to a Boltzmann function using OriginLab non-linear fitting software. Drug efficacy was defined as the percentage of maximal decrease/suppression in the wave parameter at 100 μM (highest drug concentration tested in each case). All parameter values were normalized to values obtained from cells to vehicle (VEH).

In a separate set of experiments, Ca^2+^ sparks were analyzed. The method used to obtain spark-generating cells has also been previously reported [18]. The automated detection of Ca^2+^ sparks and the measurement of temporal and spatial spark properties was carried out using the “SparkMaster” plug-in for ImageJ [27]. Spark mass was calculated with the following equation: Spark mass = spark amplitude x 1.206 x FWHM^3^ as previously reported [28]. Spark-mediated leak was calculated as the product of spark mass and spark frequency.

### Statistical analysis

The comparisons of wave incidence between the experimental and control groups were done using Fisher exact test. All other parameters were compared using non-paired Student *t*-test. Results were considered statistically significant if the P-value was less than 0.05.

## Results

### Comparison of Ca^2+^ sparks and waves in Casq2^-/-^, RyR2-R4496C^+/-^ and WT mouse myocytes

**Fig 1:**
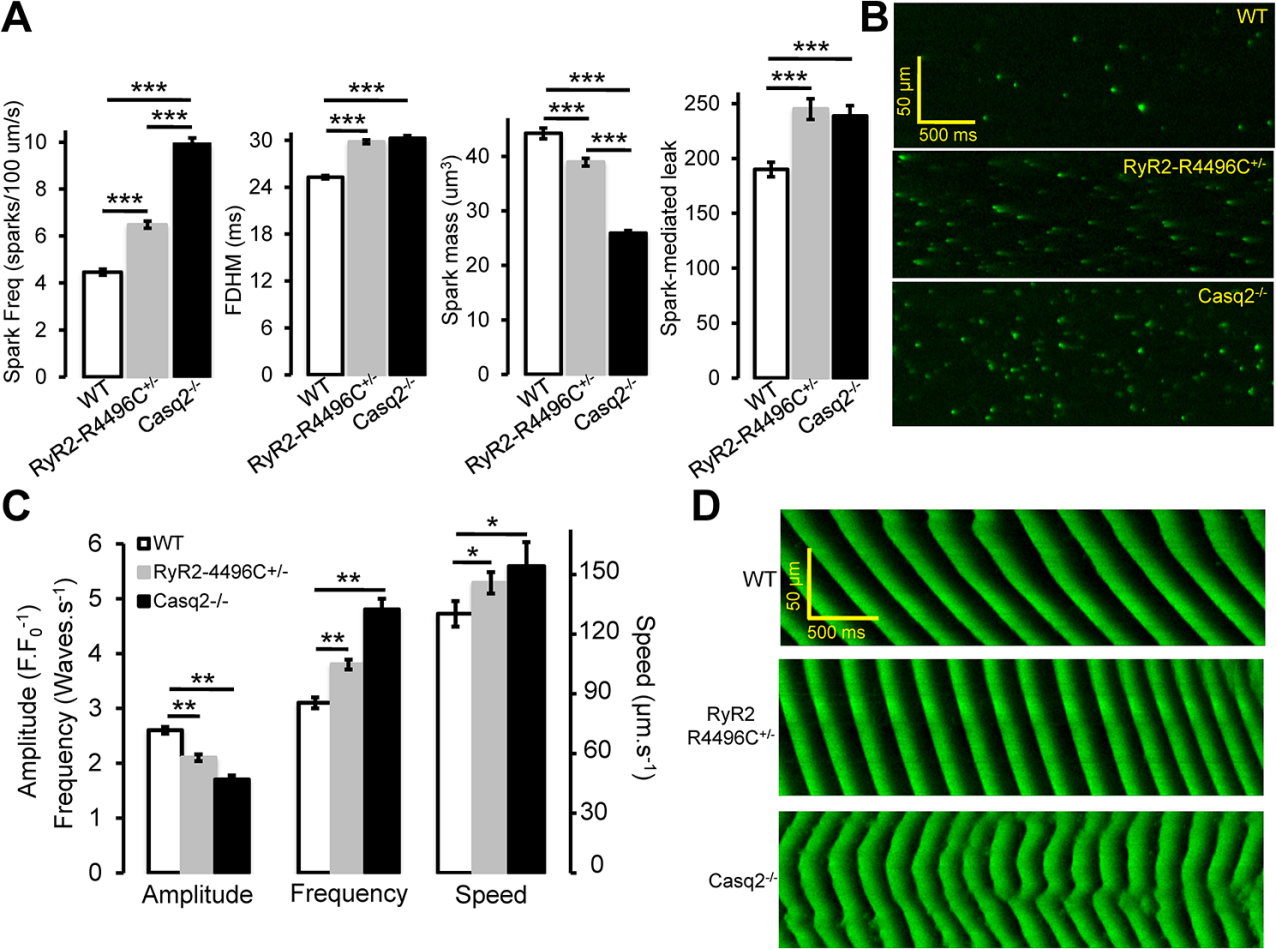
Ca^2+^ sparks and waves in permeabilized myocytes from WT, RyR2-R4496C and Casq2^-/-^ mice under control (VEH) conditions. A. Averaged spark frequency, full duration at half maximun (FDHM), spark mass and spark-mediated leak measured for each one of the groups studied. ***P<0.001, n = 35–60 cells, N = 2–3 mice/group. Results expressed as mean±SEM. B. Representative line-scans (LS) showing sparks registered in permeabilized WT, RyR2-R4496C, and Casq2^**-/-**^ myocytes in internal solution (IS) containing Fluo 4 pentapotassium salt ([EGTA]_IS_ = 0.4 mM, estimated free [Ca^**2+**^]_IS_ = 0.04 μM (Maxchelator)). C. Averaged amplitude, frequency and propagation speed of Ca^**2+**^ waves measured in WT, RyR2-R4496C and Casq2^**-/-**^ permeabilized myocytes. *P<0.05, **P<0.01. n = 30–60 cells, N = 2 mice/group. D. Representative LS of Ca^**2+**^ waves from permeabilized WT, RyR2-4496C mutant cells and Casq2^**-/-**^ myocytes in internal solution containing Fluo-4 pentapotassium salt ([EGTA]_IS_ = 0.05 mM, estimated free [Ca^**2+**^]_IS_ = 3.9 μM (Maxchelator)).

We first evaluated Ca^2+^ spark properties in permeabilized ventricular myocytes isolated from wild-type (WT), Casq2 null (Casq2^-/-^), and RyR2-RC4496C^+/-^ mice (Fig 1). Individual Ca^2+^ sparks were resolved by including 0.4 mM EGTA in the internal solution in the presence of 0.06 mM CaCl_2_ [Free [Ca^2+^] ~27 nM (Maxchelator)] and recorded in line-scan mode using confocal microscopy. Spark-mediated leak was calculated as the product between spark mass and the corresponding frequency. Consistent with an increased RyR2 channel activity, both R4496C^+/-^ and Casq2^-/-^ myocytes exhibited increased spark frequency compared to WT myocytes (Fig 1A and 1B). However, spark frequency was significantly higher in Casq2^-/-^ than in R4496C^+/-^ and WT myocytes, which may explain the more severe CPVT phenotype observed in Casq2^-/-^ compared to R4496C^+/-^ mice. Spark duration was increased to a similar extent in both CPVT models, whereas spark mass, a measure of total Ca^2+^ flux during a spark, was lowest in Casq2^-/-^, intermediated in R4496C^+/-^ and highest in WT myocytes (Fig 1A). As a result, spark mediated SR Ca^2+^ leak was increased in both CPVT mouse models.

We next tested the effect of the CPVT mutations on Ca^2+^ waves by reducing the EGTA concentration to 50 μM. Compared to WT myocytes, absence of Casq2 decreased the averaged wave amplitude, and increased both Ca^2+^ wave frequency and the speed of wave propagation along the length of the myocyte, (Fig 1C and 1D). Analogous to their Ca^2+^ spark phenotype, the Ca^2+^ wave phenotype of R4496C^+/-^ myocytes was intermediate between WT and Casq2^-/-^ cells (Fig 1C and 1D).

### Effect of RyR2 activity on efficacy and potency of FLEC, RPROP and TET against Ca^2+^ waves

**Fig 2:**
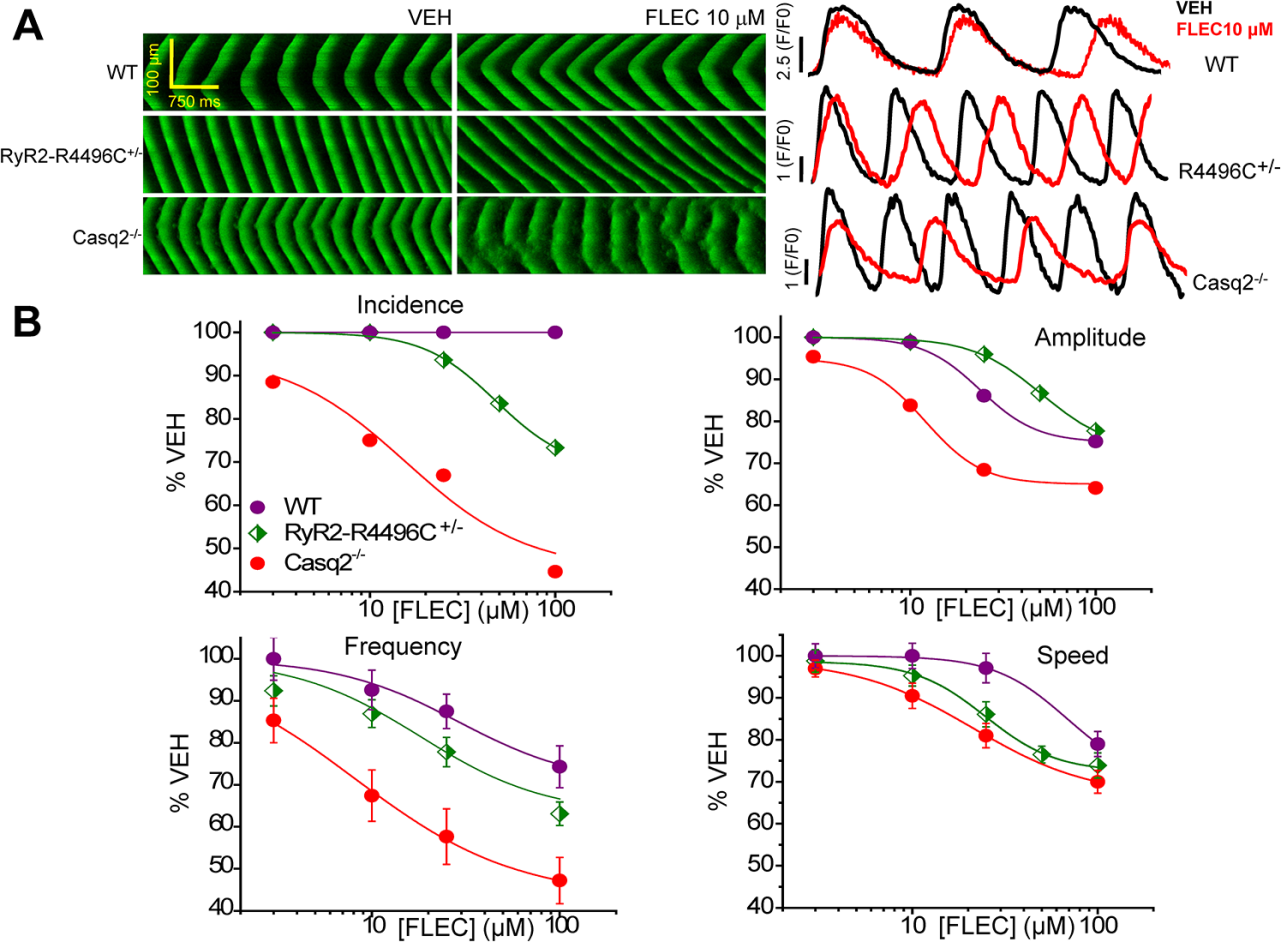
Effects of flecainide (FLEC) on Ca^2+^ wave parameters from permeabilized WT, RyR2-R4496C, and Casq2^-/-^ myocytes. A. Representative LS (left side of the panel) obtained for WT, RyR2-R4496C, and Casq2^**-/-**^ myocytes, and their corresponding averaged space LS (right side of the same panel) under control condition (VEH) and in the presence of flecainide (FLEC) 10 μM. B. Concentration-response curves (CRCs) for wave incidence, amplitude, frequency, and propagation speed as a function of the concentration of FLEC (in μM) in WT, RyR2-4496C mutant cells, and Casq2^**-/-**^ myocytes. Results are expressed as mean±SE for each concentration and each parameter. For each group studied: n = 20 cells, N = 3 mice/ condition tested.

**Fig 3:**
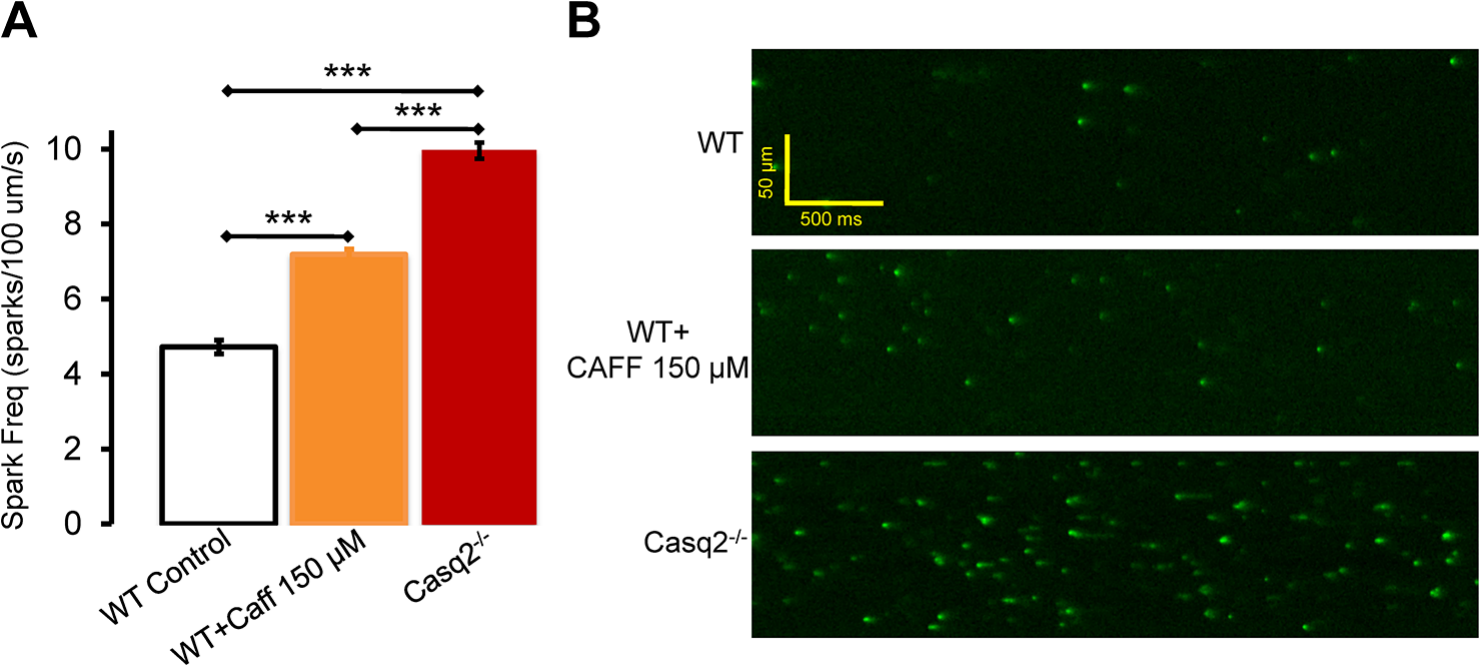
Comparison of Ca^2+^ spark frequency in WT, caffeine (CAFF) treated WT and Casq2^-/-^ myocytes. A. Average spark frequency, N = 25–70 cells/group, ***P<0.001. B. Representative LS showing sparks registered in permeabilized WT myocytes, CAFF-treated permeabilized WT myocytes, and Casq2^**-/-**^ myocytes in internal solution (IS) containing Fluo 4 pentapotassium salt ([EGTA]_IS_ = 0.4 mM, nominal [Ca^**2+**^]_IS_ = 0.06 mM, estimated free [Ca^**2+**^]_IS_ = 0.04 μM (Maxchelator)).

**Fig 4:**
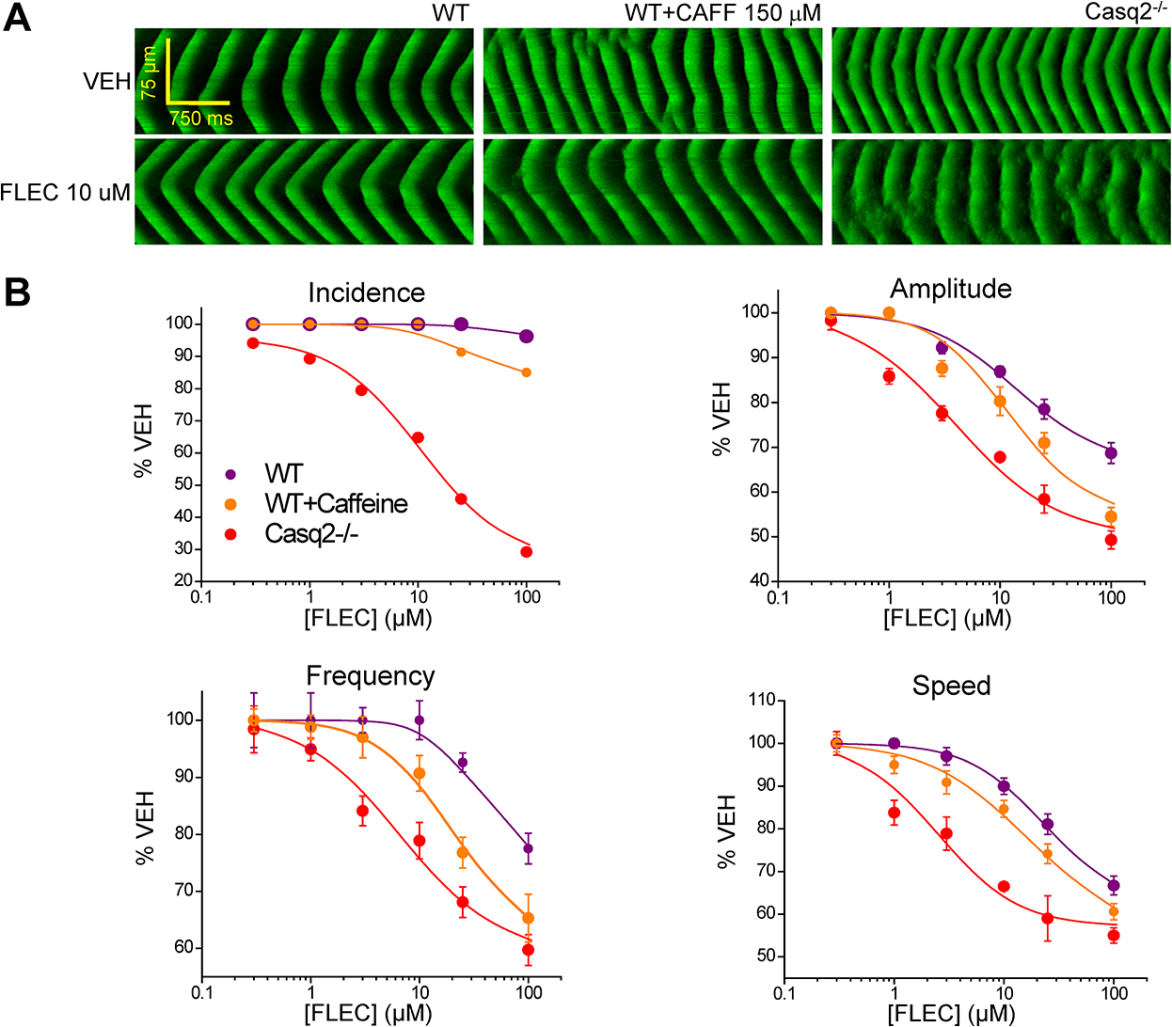
Caffeine increases potency of flecainide (FLEC) against Ca^2+^ waves. A. Representative LS of Ca^**2+**^ waves in permeabilized WT myocytes, WT cells sensitized with 150 μM caffeine (WT+CAFF 150μM) and Casq2^**-/-**^ myocytes under control condition (VEH) and in the presence of FLEC 10 μM. B. CRCs for wave parameters (incidence, amplitude, frequency, and propagation speed) as a function of the concentration of FLEC (in μM) in WT, WT+CAFF 150μM, and Casq2^**-/-**^ cells. FLEC effect on Ca^**2+**^ wave parameters is dose-dependent, with a strongest effect on Ca^**2+**^ waves in Casq2^**-/-**^ myocytes. FLEC effects on Ca^**2+**^ waves in WT cells are partially restored by sensitizing the cells with CAFF 150 μM. Mean±SE for each concentration and each parameter. For each group: n = 20 cells, N = 3 mice/condition tested.

**Fig 5:**
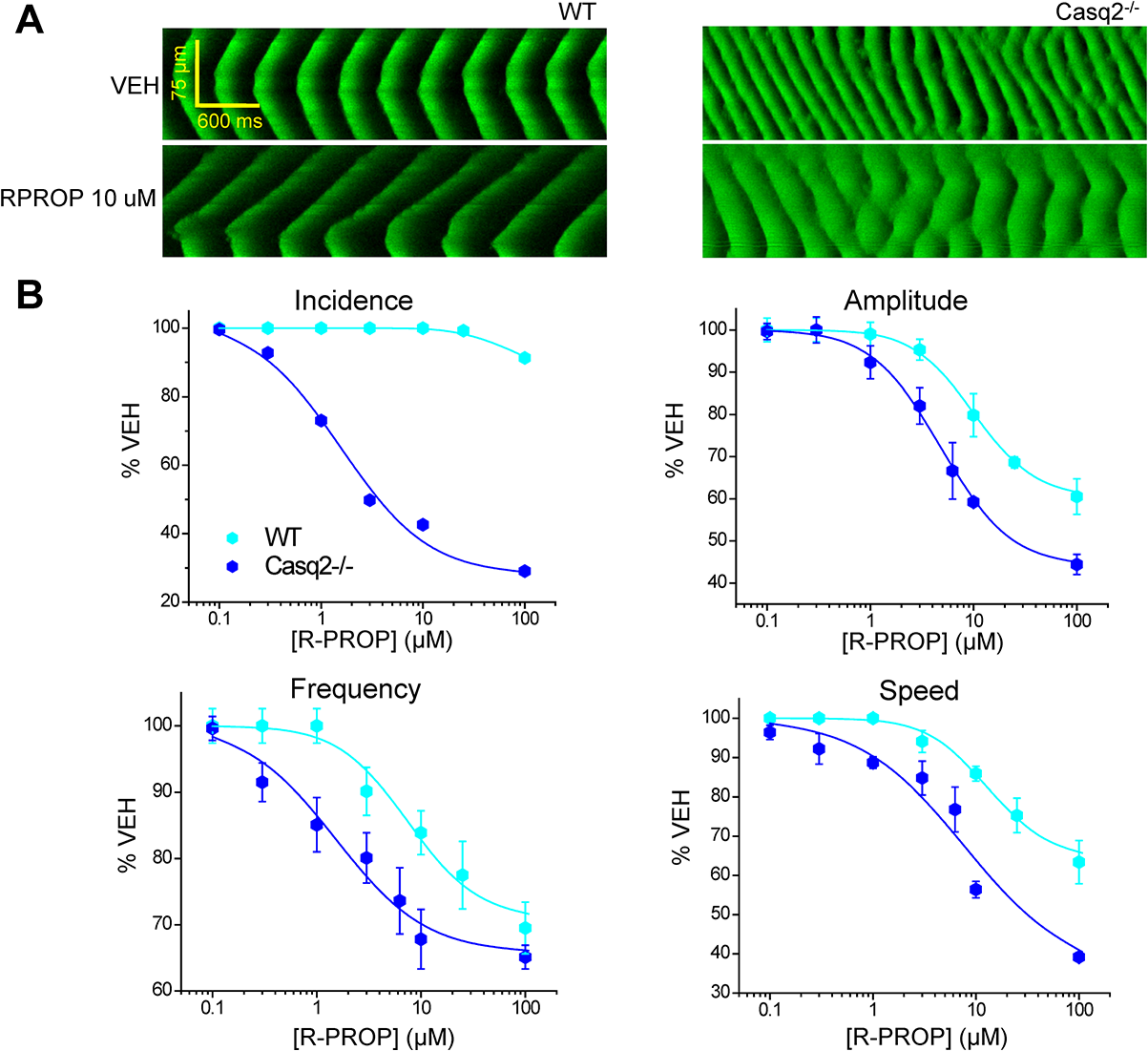
Caffeine increases potency of R-propafenone (RPROP) against Ca^2+^ waves. A. Representative line-scans of Ca^**2+**^ waves in permeabilized WT myocytes and Casq2^**-/-**^ myocytes under control condition (VEH) and in the presence of RPROP 10 μM. B. CRCs for wave parameters (incidence, amplitude, frequency, and propagation speed) as a function of the concentration of RPROP (in μM) in WT and Casq2^**-/-**^ cells. Similarly to FLEC, the effect of RPROP on the Ca^**2+**^ waves is dose-dependent, with a strongest effect on Casq2^**-/-**^ myocytes. Results expressed as mean±SE for each concentration and each parameter. For each group: n = 20–25 cells, N = 2 mice/condition tested.

The increased Ca^2+^ spark and wave parameters (Fig 1) demonstrate that both experimental CPVT models studied here (Casq2^-/-^ and RyR2-R4496C^+/-^ mice) exhibit increased intrinsic RyR2 activity. We previously showed that FLEC and RPROP inhibit RyR2 channels in the open state, increase Ca^2+^ spark frequency but reduce spark mass (“flicker” block), suppress Ca^2+^ waves and prevents CPVT in mice and humans [22]. We hypothesized that any increase in the frequency of RyR2 openings should increase FLEC and RPROP anti-arrhythmic potency and efficacy for suppressing Ca^2+^ waves. To test this hypothesis, we first studied the effects of FLEC and RPROP on Ca^2+^ wave parameters in permeabilized myocytes isolated from Casq2^-/-^, RyR2-4496C^+/-^ mutant, and WT littermates (Figs 2, 3, 4 and 5). Saponin-permeabilized myocytes from all 3 groups were exposed to increasing concentrations of FLEC and RPROP and imaged in the line-scan mode using confocal microscopy. Drug effects on wave parameters (wave incidence, amplitude, frequency and propagation speed) were studied. Concentration-response curves (CRCs) were constructed for each drug to obtain the IC_50_ (potency) and the maximum percentage of inhibition (efficacy) (Table 1). FLEC effect on Ca^2+^ wave parameters was concentration-dependent, with a maximum effect on the Ca^2+^ waves in Casq2^-/-^ myocytes (Figs 2 and 4), with an IC_50_ for Ca^2+^ wave suppression of 15.6±3.4 μM and efficacy of ~56%. FLEC had practically no effect in terms of wave suppression in WT cells (Figs 2 and 4). The effect of FLEC on Ca^2+^ waves in RyR2-R4496C^+/-^ mutant myocytes was intermediate between WT and Casq2^-/-^ (Fig 2, and Table 1). FLEC had a similar effect on the other Ca^2+^ wave parameters analyzed across the 3 groups studied, with the highest potency (lowest IC_50_) and efficacy in Casq2^-/-^ myocytes compared to the other 2 groups studied (Table 1). RPROP also exhibited a concentration-dependent effect on Ca^2+^ wave parameters (Fig 5). Similar to FLEC, RPROP affected Ca^2+^ waves in Casq2^-/-^ myocytes with higher potency (lower IC_50_) and efficacy compared to its effect on the other 2 groups studied (RyR2-R4496C^+/-^ and WT cells, Table 1).

**Table 1:** Potency (IC_50_, expressed in μM) and efficacy (defined as maximum drug effect measured at 100 μM) of all the drugs tested in the different mouse groups.

	Wave Suppression		Wave Amplitude		Wave frequency		Speed of Propagation	
Drugs	Potency	Efficacy	Potency	Efficacy	Potency	Efficacy	Potency	Efficacy
WT FLEC	>100	0	23.8±1.4	22.1	29.2±2.1	22.5	54.8±7.2	33.3
WT+Caff FLEC	27.1±3	15	11.5±3.4	45.5	19.2±1.2	34	15.9±3.5	39.4
RyR2-R4496C^+/-^ FLEC	47.8±5	26.7	18.6±0.5	26.4	19.2±6.5	35.8	19.9±2.4	36.1
Casq2^-/-^ FLEC	15.6±3.4	56	12±0.6	35	9.1±0.6	41	13.5±0.2	32
WT RPROP	54.3±1.4	8.7	19.6±1.3	33.5	6.3±3.1	30.5	22.1±4.6	36.4
RyR2-R4496C^+/-^ RPROP	21.2±0.7	52	15.7±4.1	49.5	3±0.7	14	20.3±4.1	32.14
Casq2^-/-^ RPROP	1.9±0.4	69	5.1±0.6	53	2.7±0.5	34	10.5±0.9	58
WT TET	N/A	N/A	N/A	N/A	22.1±3.3	53.6	19.8±5.6	41.2
WT+Caff TET	N/A	N/A	N/A	N/A	17.1±1.9	53.1	21.6±3.4	34.6
Casq2^-/-^ TET	N/A	N/A	N/A	N/A	28.1±2.6	44.44	21.6±2.2	54.6

N = 20–25 cells/condition tested. All measurements are relative to measurements obtained in cells exposed to VEH.

Because both Casq2^-/-^ and RyR2-4496C^+/-^ mutant myocytes exhibit higher frequency of sparks and spontaneous Ca^2+^ waves, we hypothesized that an increased RyR2 opening frequency would make myocytes more sensitive to open-channel blockers like FLEC and RPROP. To directly test this hypothesis, we first studied the effect of caffeine (CAF) 150 μM on the spark frequency of permeabilized WT ventricular myocytes. After 5 min incubation in CAFF 150 μM, CAFF-treated cells exhibited a significantly increased spark frequency compared to the spark frequency observed in vehicle-treated WT cells (Fig 3, panels A and B). Note that spark frequency in CAFF-treated WT cells was significantly lower than the spark frequency observed in permeabilized Casq2^-/-^ myocytes (Fig 3). Next, we studied the effect of FLEC on Ca^2+^ waves in CAFF-treated WT myocytes (Fig 4). After incubating the WT myocytes with CAFF (150 μM) for 5 min, CRCs for FLEC were obtained and compared to those obtained in control WT and Casq2^-/-^ myocytes (Fig 4 and Table 1). Exposure of WT cells to CAFF increased Ca^2+^ wave frequency, partially resembling that of Casq2^-/-^ myocytes (Fig 4, panel A center). CAFF also made WT myocytes responsive to FLEC, increasing the apparent potency and efficacy of wave inhibition by FLEC (Fig 4 and Table 1). In CAFF-sensitized WT myocytes, FLEC values of IC_50_ and efficacy for Ca^2+^ wave suppression and the other wave parameters were on average intermediate between the values previously obtained in Casq2^-/-^ and non-sensitized WT cells (Table 1). The increased sensitivity to FLEC observed in CAFF sensitized WT cells further support our hypothesis that increased RyR2 open probability contributes to determine the potency and efficacy of antiarrhythmic open channel blockers like FLEC.

**Fig 6:**
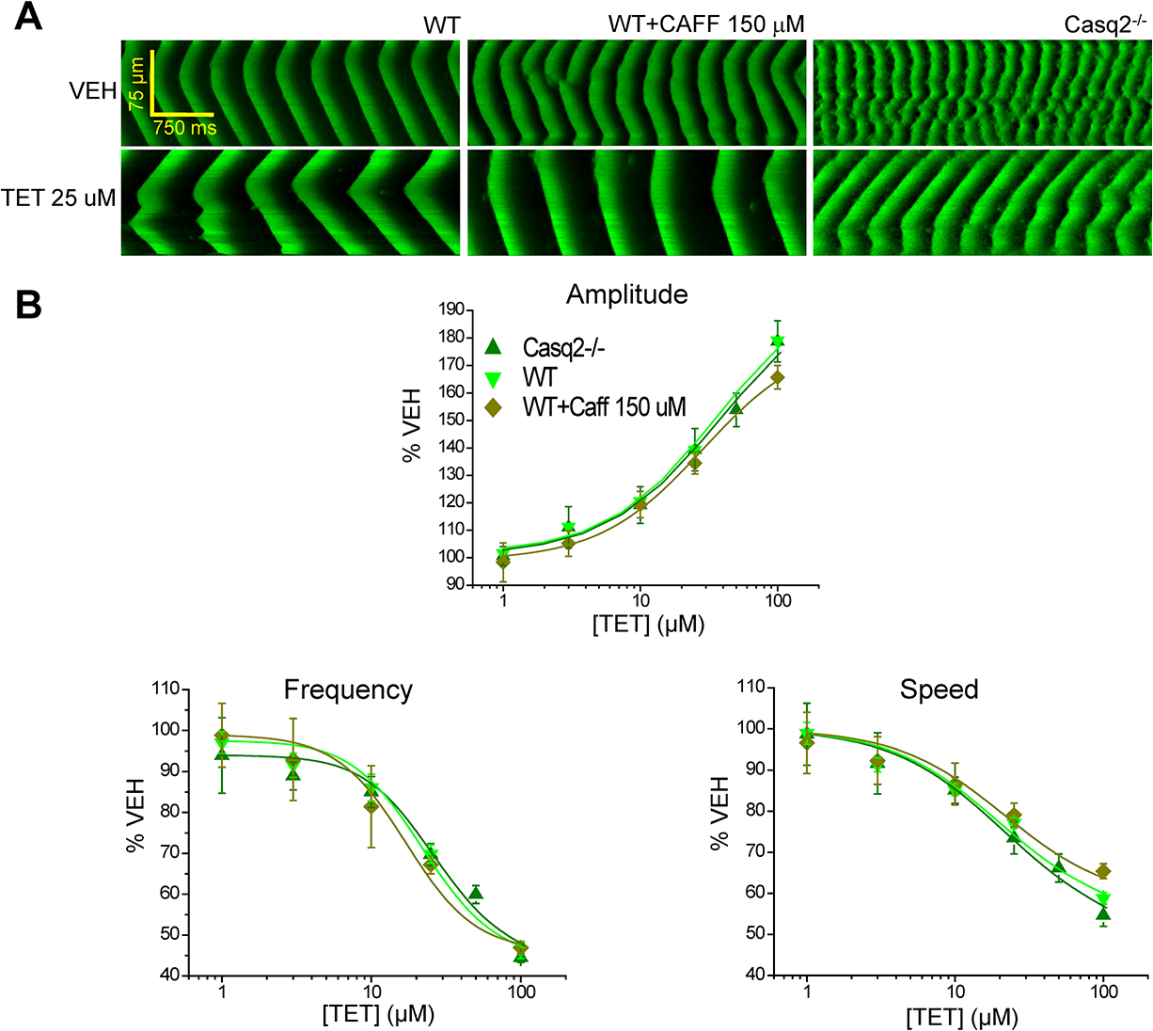
Caffeine has no effect on potency of tetracaine (TET) against Ca^2+^ waves. A. Representative LS of Ca^**2+**^ waves in permeabilized WT myocytes, WT+CAFF 150μM, and Casq2^**-/-**^ myocytes under control condition (VEH) and in the presence of tetracaine (TET) 25 μM. B. CRCs for wave parameters (incidence, amplitude, frequency, and propagation speed) as a function of TET concentration (in μM) in WT, WT+CAFF 150μM, and Casq2^**-/-**^ cells. Mean±SEM is depicted for each concentration and each parameter. For each group: n = 20–25 cells, N = 2–3 mice/ condition tested.

We next examined the effect of tetracaine (TET) on Ca^2+^ wave parameters in WT, CAFF-sensitized WT, and Casq2^-/-^ myocytes (Fig 6, panels A and B). TET is an amino-ester classically used as a local anesthetic that unlike FLEC blocks RyR2 independently of the state of the channel (state-independent RyR2 blocker) [29]. The corresponding CRCs obtained for TET in each group studied are presented in Fig 6. The IC_50_s and efficacies for the 3 groups studied are shown in Table 1. In contrast to what was observed with FLEC and RPROP, TET did not suppress Ca^2+^ waves, but rather increased the incidence of Ca^2+^ waves. The effect of TET on wave frequency, wave amplitude and wave speed was dose-dependent, but was similar across all three groups tested (Fig 6). Furthermore, incubation of WT cardiomyocytes with CAFF 150 μM also did not modify the potency and efficacy of TET on Ca^2+^ wave parameters (Fig 6 and Table 1).

### Effect of RPROP and TET on Ca^2+^ sparks and waves in rabbit ventricular myocytes

**Fig 7:**
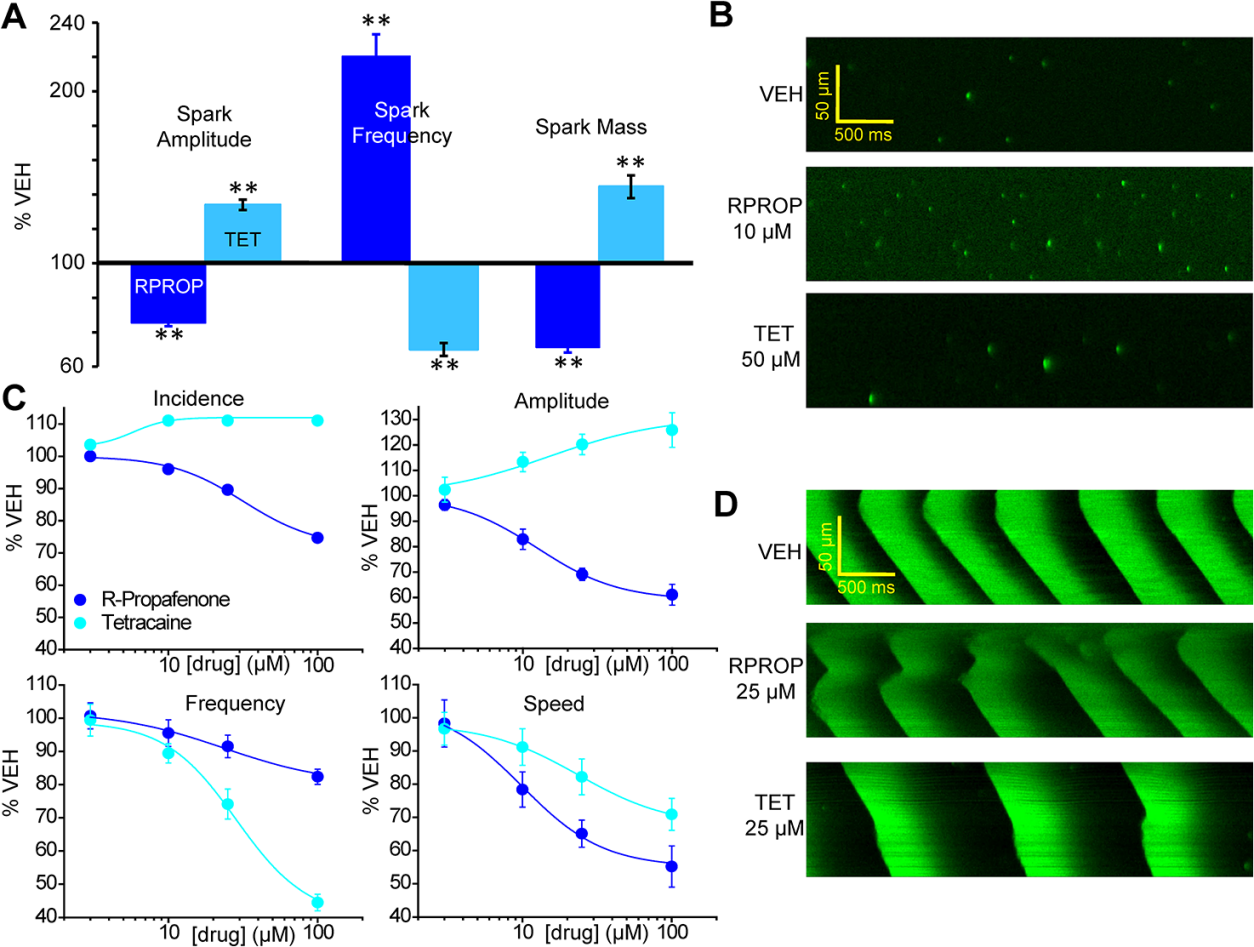
Effect of RPROP and TET on Ca^2+^ sparks and waves in permeabilized rabbit ventricular myocytes. A. Bar representation (left side of the panel) of averaged Ca^**2+**^ spark parameters (amplitude, frequency and mass) measured in permeabilized rabbit ventricular myocytes under control conditions (VEH) and in the presence of RPROP 10 μM and TET 50 μM. **P<0.01. n = 30–40 cells, N = 3 rabbits/condition tested. B Representative LS for each condition. C CRCs for RPROP and TET for Ca^**2+**^ wave parameters. n = 30 cells, N = 3 rabbits/condition tested. D. Representative LS for VEH, RPROP 25 μM and TET 25 μM.

Due to their higher intrinsic RyR2 opening frequency [30], we hypothesized that rabbit ventricular myocytes would be more sensitive to an open channel blocker like RPROP than mouse WT myocytes. To test this hypothesis, we first measured Ca^2+^ sparks in saponin-permeabilized rabbit ventricular myocytes at baseline and examined the effect of RPROP (10 μM) on spark parameters (Fig 7A and 7B), a concentration that has no effect on mouse WT myocytes (Fig 5). As expected, permeabilized rabbit ventricular myocytes exhibited a significantly higher averaged spark frequency (6.3±0.45 sparks/100 μm/s) compared to WT mouse myocytes under control conditions (Fig 1). Next, we studied the effects of RPROP and TET in permeabilized rabbit myocytes. Analogous to its effect in Casq2 KO myocytes [18, 19], RPROP significantly *decreased* Ca^2+^ spark amplitude and spark mass while *increasing* spark frequency (Fig 7, panels A and B). In contrast, TET 50 μM significantly *decreased* spark frequency, and *increased* spark amplitude and mass (Fig 7, panels A and B). We next investigated the effects of both drugs on Ca^2+^ waves. CRCs were obtained for RPROP (open-channel blocker) and TET (non-state dependent RyR2 channel blocker) and IC_50_ and efficacy calculated for the each wave parameter (Fig 7, panels C and D, and Table 2). RPROP significantly reduced the incidence of Ca^2+^ waves in rabbit myocytes (Table 2) whereas RPROP had no effect on wave incidence in mouse WT myocytes (Table 1). In addition, RPROP exhibited a higher potency (lower IC_50_) and efficacy in all other wave parameters analyzed in rabbit myocytes compared to mouse WT cells (compare Tables 1 and 2). These results are in agreement with the results obtained with Casq2^-/-^ and RyR2-R4496C^+/-^ myocytes, and further support our hypothesis that increased RyR2 open probability determines the potency and efficacy of open channel blockers for inhibiting Ca^2+^ waves.

**Table 2:** Potency (IC_50_, expressed in μM) and efficacy (defined as maximum drug effect measured at 100 μM) of RPROP and TET in permeabilized rabbit ventricular myocytes.

	Wave Suppression		Wave Amplitude		Wave frequency		Speed of Propagation	
Rabbit myocytes	Potency	Efficacy	Potency	Efficacy	Potency	Efficacy	Potency	Efficacy
RPROP	31.9±1.6	25.4	11.5±0.3	39	21.5±2.1	20.5	10.2±0.5	45
TET	N/A	N/A	N/A	N/A	29.6±3.4	44	23.7	29

## Discussion

### Controversy over the importance of Na^+^ versus RyR2 block for antiarrhythmic drug efficacy in CPVT

The clinical management of CPVT has been improved in recent years with the introduction of FLEC, a drug that effectively suppresses Ca^2+^ waves *in vitro* [21] and prevents CPVT in humans [22, 31, 32]. However, the pharmacological mechanisms that determine the clinical effectiveness of FLEC in the management of CPVT remain controversial. Based on our studies of FLEC in single RyR2 channels and isolated Casq2^-/-^ myocytes, we proposed that Na^+^ and RyR2 channel block have additive effects and together are contributing for FLEC’s striking clinical efficacy in CPVT [22]. The importance of RyR2 block in CPVT is further supported by studies indicating that carvedilol analogs, which are effective against CPVT in mouse models, exhibit RyR2 blocking properties [33]. On the other hand, Sikkel et al. recently reported that RyR2 block did not contribute to Ca^2+^ wave inhibition by FLEC in healthy rat myocytes [24]. Liu et al. reported that FLEC did not modulate Ca^2+^ spark rate in myocytes from RyR2-R4496C^+/-^ mice, another CPVT model [23]. Bannister et al. (2015) reported similar results in hRyR2 single channels and healthy permeabilized rat myocytes [34]. Based on these results, these groups concluded that FLEC acts primarily as a Na^+^ channel blocker in CPVT. One purpose of the current study was to address this controversy by examining the effect of RyR2 activity on the potency and efficacy of FLEC and RPROP against Ca^2+^ waves in ventricular myocytes after membrane permeabilization, which excludes any possible effects of Na^+^ channel blockade by FLEC on Ca^2+^ waves. We have previously shown that Na channel block has no effect on Ca waves in permeabilized myocytes. Furthermore, we hypothesized that differences in RyR2 activity (i.e., lower activity in healthy rat myocytes compared to CPVT myocytes) determine the potency of open-state RyR2 blockers such as FLEC against Ca^2+^ waves in cardiomyocytes and could help explain the differences between our results and that of other groups. Consistent with that hypothesis, we found that differences in the rate of Ca^2+^ sparks, an estimate of RyR2 activity, between WT, CAFF-treated WT, Casq2^-/-^ and RyR2-4496C^+/-^ myocytes directly predicted the Ca^2+^ wave blocking potency of FLEC and RPROP but not that of the closed channel blocker TET. Also consistent with that hypothesis that RyR2 activity determines FLEC potency, activating Ca^2+^ sparks with low dose of CAFF rendered WT myocytes more susceptible to Ca^2+^ wave inhibition by FLEC. Finally, WT rabbit myocytes with intrinsically higher RyR2 Po (higher spark frequency than WT cells) are more sensitive to FLEC and PROP than WT mouse myocytes. Taken together, these results demonstrate that Ca^2+^ spark rate and hence intrinsic RyR2 channel activity is an important predictor for the potency of RyR2 open channel blockers FLEC and RPROP against Ca^2+^ waves, which may help explain why the contribution of RyR2 block to Ca^2+^ wave inhibition by FLEC was not as evident in studies of healthy rat ventricular myocytes [24, 34] or RyR2-R4496C^+/-^ ventricular myocytes [23], which both exhibit lower intrinsic RyR2 activity compared to Casq2^-/-^ myocytes. Further supporting our hypothesis are reports that flecainide was highly effective against Ca waves in RyR2-R4496C^+/-^ Purkinje cells, which have intrinsically higher spark frequency than RyR2-R4496C^+/-^ ventricular myocytes [35].

### RyR2 “use-dependence” of Ca^2+^ wave inhibition

In essence, we report that open state blockers FLEC and RPROP exhibit RyR2 “use-dependence” in their action against Ca^2+^ waves, which was not the case for the state-independent blocker TET: Increasing RyR2 activity increases the potency of FLEC and RPROP action. Interestingly, open channel block and use-dependence (i.e., increasing block with repetitive channel activation) is also a typical feature of FLEC action on cardiac Na^+^ channels [36]. For Na^+^ channels, the underlying mechanism of FLEC use-dependence is well-established: FLEC requires Na^+^ channel opening to gain access to the inactivated state [37], to which FLEC then preferentially binds [38]. What are the possible mechanisms responsible for the apparent use-dependence of FLEC against Ca^2+^ waves reported here? It seems unlikely that FLEC binds with higher affinity to RyR2 in the open configuration based on our previous studies of single RyR2 channels in artificial bilayers: FLEC block was less potent at pCa4 when RyR2 channels were fully activated compared to pCa7 when RyR2 activity was much lower [22]. A more likely explanation is that the phenomenon of use dependence of open channel blockers occurs at the level of the Ca^2+^ spark that triggers a propagated Ca^2+^ wave. The likelihood of a local Ca^2+^ release for triggering a Ca^2+^ wave depends on two main factors: the amount of Ca^2+^ released (i.e., Ca^2+^ spark mass) and the Ca^2+^ sensitivity of a neighboring RyR2 cluster (i.e., intrinsic RyR2 activity). Increasing RyR2 activity will promote Ca^2+^ waves, but at the same time reduce SR Ca^2+^ content and hence Ca^2+^ spark mass due to the increased SR Ca^2+^ leak until a new steady state is reached [39]. We have shown that the mechanism of FLEC action is an open RyR2 channel blocker, reducing both the mean open burst duration and burst Po of RyR2 [21]. The reduced RyR2 burst duration decreases RyR2 Ca^2+^ flux per unit time and hence decreases Ca^2+^ spark mass. The reduction in Ca^2+^ spark mass, in turn, reduces the likelihood of activating neighboring RyR2 clusters and therefore propagation of Ca^2+^-induced Ca^2+^ release in the form of Ca^2+^ waves [21]. Hence, the most likely explanation for the apparent use dependence of FLEC and RPROP is that increasing RyR2 activity reduces SR Ca^2+^ content. Although more frequent, the resulting Ca^2+^ waves are smaller and therefore easier to break up by drugs that reduce Ca^2+^ spark mass. Consistent with that hypothesis is our experimental observation that FLEC and RPROP break up propagated Ca^2+^ waves into small non-propagated wavelets and macro sparks [18]. Furthermore, pretreatment with low dose CAFF, which increases spark frequency and SR Ca^2+^ leak and reduces SR Ca^2+^ content, also increased the potency of FLEC and RPROP.

### Implications for drug development of Ca^2+^ wave inhibitors and study limitations

What are the implications of use-dependence of Ca^2+^ wave block for developing drugs against Ca^2+^ wave triggered arrhythmias? For one, drugs like FLEC may act preferentially on diseased myocytes with higher RyR2 activity than healthy myocytes. For example, the increased frequency in both Ca^2+^ sparks and waves observed in myocytes from the two CPVT models studied here are in agreement with previous results reported for these and other CPVT mouse models [18, 37, 40, 41]. Increased RyR2 activity and Ca^2+^ leak are also frequently found in myocytes isolated from animal models of heart failure [42, 43], and RyR2 hyperactivity likely contributes to ventricular arrhythmia risk [40]. While Class IC agents such as FLEC and RPROP are contraindicated in ischemia or heart failure due to their Na^+^ channel blocking properties [41], targeting RyR2 with agents such as carvedilol that exhibit open channel RyR2 block and hence use-dependence against Ca^2+^ waves appears a promising new paradigm for finding better anti-antiarrhythmic medicines [44].

We would like to emphasize that this study was not designed to address the clinical relevance of RyR2 block for the antiarrhythmic efficacy of flecainide and propafenone in patients. Permeabilized myocytes are a highly reduced system and whether the IC50 concentrations identified in our study are applicable to the clinical care is not known.

## Conclusions

Our study demonstrates that RyR2 activity determines the potency and efficacy of the open-state blockers FLEC and RPROP for suppressing arrhythmogenic Ca^2+^ waves in cardiomyocytes from WT mice, CPVT mouse models and rabbits. The use-dependence of Ca^2+^ wave inhibition reported here may represent a promising new paradigm for future antiarrhythmic drug development.

## Supporting Information

S1 ARRIVE Guidelines Checklist Animal Research: Reporting of In Vivo Experiments (ARRIVE) guidelines checklist. (PDF)
